# The association of ghrelin rs26311 and rs27647 polymorphisms and mRNA expression with preeclampsia susceptibility and severity—A case–control study

**DOI:** 10.1590/1806-9282.20231638

**Published:** 2024-09-13

**Authors:** Farzaneh Montazerifar, Saeedeh Salimi, Rasul Taghvaeefar, Mansour Karajibani, Marzieh Ghasemi, Mehrnaz Mehrabani, Mahnaz Rezaei

**Affiliations:** 1 Zahedan University of Medical Sciences, School of Medicine, Department of Nutrition – Zahedan, Iran.; 2 Zahedan University of Medical Sciences, Pregnancy Health Research Center – Zahedan, Iran.; 3 Shahid Beheshti University of Medical Sciences, School of Medicine, Department of Clinical Biochemistry – Tehran, Iran.; 4 Behbahan Faculty of medical Sciences – Behbahan, Iran.; 5 Zahedan University of Medical Sciences, Health Promotion Research Center – Zahedan, Iran.; 6 Zahedan University of Medical Sciences, School of Medicine, Department of Obstetrics and Gynecology – Zahedan, Iran.; 7 Kerman University of Medical Sciences, Institute of Basic and Clinical Physiology Sciences, Physiology Research Center – Kerman, Iran.; 8 Zahedan University of Medical Sciences, School of Medicine, Department of Clinical Biochemistry – Zahedan, Iran.

**Keywords:** Gene expression, Ghrelin, Polymorphism, Preeclampsia, Severity

## Abstract

**OBJECTIVE:**

Ghrelin is an adipokine the placenta generates to control the maternal metabolic adaptation to pregnancy. It causes different pregnancy complications like preeclampsia (PE). Therefore, the aim of this study was to assess the association between ghrelin mRNA expression and rs26311 and rs27647 polymorphisms and PE development.

**METHODS:**

In total, 156 PE women (including 97 patients with mild PE and 59 patients with severe PE) and 152 healthy controls were recruited in this case–control study during 2019–2020. All participants with other diseases have been excluded from both groups. The ghrelin expression was analyzed with real-time PCR, and ghrelin variants were examined using the RFLP-PCR method.

**RESULTS:**

The maternal and placental ghrelin rs27647 and rs26311 variants were unrelated to PE susceptibility. Haplotype analyses showed no significant difference between the four haplotypes and PE. No relationship was revealed between rs27647 polymorphism and severe PE. However, the results indicated a relationship between rs27647 and severe PE compared to mild PE and controls. Therefore, the rs27647 variant was associated with severe PE compared to mild PE in codominant, recessive, and log-additive models and controls in codominant, dominant, recessive, and log-additive models. The placental ghrelin mRNA expression declined in PE women compared to controls (0.67-fold), but the difference was insignificant (p=0.263). No significant difference was found between various genotypes of rs27647 and rs26311 polymorphisms concerning ghrelin mRNA expression.

**CONCLUSION:**

The maternal and placental ghrelin polymorphisms, rs27647 and rs26311, showed no effect on PE. However, the rs27647 variant was associated with severe PE.

## INTRODUCTION

Preeclampsia (PE) is a multi-systemic complication that is specific to pregnancy^1,2^. This syndrome is associated with fetal, infant, and maternal mortality. The incidence of PE is approximately 4.6% of all pregnant women worldwide^3^. It is characterized by proteinuria (≥300 mg/24 h), hypertension (blood pressure ≥140/90), and an excessive systemic inflammatory response of the mother from the 20th week of pregnancy onward^4^. PE affects various organs such as the peripheral vasculature, central nervous system, heart, liver, and kidneys and thus can be the main cause of maternal and neonatal morbidity and mortality^5^. It is believed that the combination of different factors contributes to the onset of this disorder. This problem is affected by different factors and various causes, including immunogenic, genetic, and environmental factors that could affect its pathogenesis.

The main mechanisms of PE pathogenesis are thought to be inappropriate immune response, placental ischemia, insufficient trophoblast invasion and placental development, endothelial dysfunction, and oxidative stress. However, the accurate etiology is not yet known^6^. Ghrelin is a 28-amino acid hormone and a potent growth hormone stimulant, mainly produced by the gastrointestinal tract, especially the stomach^7^. The ghrelin hormone controls various physiological functions such as body temperature, gastric motility and secretion, circulatory system, stress responses, growth hormone (GH) secretion, and feeding, and its receptor is expressed in central and peripheral organs^8^. Evidence showed a higher ghrelin concentration in pregnant women with hypertension compared to normal pregnant women. Moreover, a significant relationship between ghrelin levels and systemic blood pressure has been observed^9,10^. The other study revealed lower ghrelin levels in the maternal serum of preeclamptic women compared to controls^11^. Moreover, there was a correlation between serum ghrelin concentration and decreased blood pressure in PE women^12^.

There are several polymorphisms in the ghrelin gene including rs26311 and rs27647 variants and their effects on different diseases such as cerebral infarction and metabolic syndrome have been described^13,14^.

Regarding the effect of genetic variation on PE susceptibility, several investigations have been conducted to investigate the association between the different genetic variants and PE susceptibility. However, there is no report regarding the effects of ghrelin polymorphisms on PE. Therefore, in the present study, we investigated the association of ghrelin polymorphisms (rs26311 and rs27647) and ghrelin mRNA expression in pregnant women with PE compared to healthy pregnant women.

## METHODS

### Sample collection

This case–control includes 156 cases of PE and 152 healthy controls referred to Ali ebn Abitaleb Hospital in Zahedan, Iran. All subjects during 2019–2020 were recruited for the study. The PE and control groups were matched for age and BMI. The ethical committee of Zahedan University of Medical Sciences approved the study protocol (IR.ZAUMS.REC.1399.428) and the written informed consent form was signed by all the participants.

Diagnosis of PE is the presence of blood pressure (systolic blood pressure ≥140 mmHg or diastolic blood pressure ≥90 mmHg on at least two occasions 6 h apart) and proteinuria (≥300 mg in a 24-h urine collection or a score of ≥1 on at least one dipstick measurement) after the 20th week of pregnancy according to the definition of International Society of Hypertension in Pregnancy^15^. Severe PE was diagnosed with severe hypertension (SBP ≥160 mmHg or DBP ≥110 mmHg) or severe proteinuria (≥5 g protein in a 24-h urine collection) on two occasions at least 4 h apart^16^. The control group was selected from healthy pregnant women in the same hospital. All pregnant women with twin or multiple pregnancies and patients with diabetes, kidney, liver disease or systemic diseases, lupus, and the like, or fetuses with hydatidiform moles and fetal hydrops were ruled out of the study.

In total, 2 mL of blood was taken from each subject on EDTA and kept in freezer at –20°C until extraction. After collecting all samples, 500 μL of whole blood was separated from each of them, and its DNA was extracted by the salting out method. The placental tissues of 53 PE women and 46 non-PE pregnant women were separated after the delivery. All of the tissues were eluted with phosphate-buffered saline to remove the maternal blood and stored at -80°C for further analysis.

### Genetic analysis

The genotyping of both polymorphisms was determined using the PCR-RFLP technique^17^. The fragments containing the ghrelin polymorphisms were amplified using forward (5-CACAGCAACAAAGCTGCACC-3) and reverse (5-AAGTCCAGCCAGAGCATGCC-3) primers for rs27647 (annealing temperature=61°C) and forward (5-GCGTAGATCTTCCACCTCCA-3) and reverse (5-CGTTGTTTCCCATGTGCTGT-3) primers for rs26311 (annealing temperature=60°C). The PCR products were digested with *DraI* and *BcnI* enzymes, respectively. The digested fragments were electrophoresed on 2% agarose gel containing Safe stain. The size of the digested fragments was GG: 929bp, AG: 929+664+265bp, and AA: 664+265bp for rs27647 and CC: 289bp, GC: 289+228+61bp, and GG: 228+61bp for rs26311 polymorphisms.

The placental RNA was isolated using RNX-Plus (Sinaclon, Iran). According to the supplier's instruction, the cDNA synthesis was done using the PrimeScript1st strand cDNA Synthesis Kit (Takara Bio, Shiga, Japan).

The mRNA expression of the ghrelin gene was done by real-time PCR as previously described^18^. The mRNA expression was assayed using forward (5-GAGGATGAACTGGAAGTCCG-3) and reverse (5-CATTTATTCGCCTCCTGAGC-3) primers for ghrelin and forward (5-ACAACTTTGGTATCGTGGAAGG-3) and reverse (5-GCCATCACGCCACAGTTTC-3) primers for GAPDH genes (annealing temperature=55°C). The size of the amplified products was 264 and 101bp, respectively. The relative ghrelin mRNA expression was assayed by the 2^-ΔΔCt^ method.

### Statistical analysis

SPSS version 23 software was used for statistical tests. The descriptive statistics were examined by the Fisher exact test, T-test, or Mann-Whitney test whenever appropriate. Differences between the genotypic and allelic distributions of the study groups and subgroups were calculated using SNPStats (http://bioinfo.iconcologia.net/snpstats/start.htm) in various genetic models by calculating the odds ratio (OR) and their CI of 95% (95%CI).

## RESULTS

There was no significant difference in maternal age and BMI between the control and PE groups. As expected, the declining birth weight of neonates and gestational age resulted in PE women. The primiparity was more frequent in the PE group (42.3 vs. 29%, p=0.017).

The maternal ghrelin rs27647 AG and GG genotypes were more frequent in PE women, but the differences were insignificant. No relationship was observed between maternal and placental rs27647 polymorphism and PE in codominant, dominant, recessive, overdominant, and log-additive models. No relationship was found between maternal and placental ghrelin rs26311 variants and PE in all genetic models (Tables 1 and 2).

**Table 1:** Allelic and genotypic frequency of maternal ghrelin rs27647 and rs26311 polymorphisms in preeclampsia women and control group.

		Control (n=152)	PE (n=156)	p-value	OR (95%CI)
rs27647					
	AA, n (%)	95 (62.5)	87 (55.8)		1
	AG, n (%)	46 (30.3)	53 (34)	0.42	1.26 (0.77–2.05)
	GG, n (%)	11 (7.2)	16 (10.2)		1.59 (0.70–3.61)
	Dominant (AG + GG vs. AA)			0.23	1.32 (0.84–2.08)
	Recessive (GG vs. AA + AG)			0.35	1.46 (0.66–3.27)
	Overdominant (GG +AA vs. AG)			0.49	1.19 (0.73–1.91)
	Log-additive (GG vs. AG vs. AA)			0.19	1.26 (0.89–1.78)
Allele					
	A, n (%)	236 (78)	227 (73)		1
	G, n (%)	68 (22)	85 (27)	0.16	1.30 (0.90–1.88)
rs26311					
	GG, n (%)	95 (62.5)	105 (67.3)		1
	GC, n (%)	45 (29.6)	43 (27.6)	0.52	0.86 (0.52–1.43)
	CC, n (%)	12 (7.9)	8 (5.1)		0.60 (0.24–1.54)
	Dominant (GC+CC vs. GG)			0.38	0.81 (0.51–1.29)
	Recessive (CC vs. GG + GC)			0.32	0.63 (0.25–1.59)
	Overdominant (GG+CC vs. GC)			0.69	0.90 (0.55–1.48)
	Log-additive (CC vs. GC vs. CC)			0.28	0.82 (0.56–1.18)
Allele					
	G, n (%)	235 (77)	253 (81)		-
	C, n (%)	69 (23)	59 (19)	0.28	0.79 (0.54–1.17)

**Table 2:** Allelic and genotypic frequency of placental ghrelin rs27647 and rs26311 polymorphisms in preeclampsia women and control group.

		Control (n=46)	PE (n=53)	p-value	OR (95%CI)
rs27647					
	AA, n (%)	28 (60.9)	33 (62.3)		1
	AG, n (%)	14 (30.4)	15 (28.3)	0.97	1.10 (0.45–2.67)
	GG, n (%)	4 (8.7)	5 (9.4)		0.94 (0.23–3.85)
	Dominant (AG + GG vs. AA)			0.89	1.06 (0.47–2.39)
	Recessive (GG vs. AA + AG)			0.90	0.91 (0.23–3.63)
	Overdominant (GG +AA vs. AG)			0.82	1.11 (0.47–2.64)
	Log-additive (GG vs. AG vs. AA)			0.96	1.02 (0.56–1.85)
Allele					
	A, n (%)	70 (76)	81 (76)		1
	G, n (%)	22 (24)	25 (24)	1	1.02 (0.53–1.96)
rs26311					
	GG, n (%)	29 (63)	36 (67.9)		1
	GC, n (%)	13 (28.3)	14 (26.4)	0.80	1.15 (0.47–2.83)
	CC, n (%)	4 (8.7)	3 (5.7)		1.66 (0.34–7.99)
	Dominant (GC+CC vs. GG)			0.61	1.24 (0.54–2.85)
	Recessive (CC vs. GG + GC)			0.56	1.59 (0.34–7.49)
	Overdominant (GG +CC vs. GC)			0.84	1.10 (0.45–2.66)
	Log-additive (CC vs. GC vs. CC)			0.53	1.23 (0.65–2.33)
Allele					
	G, n (%)	71 (77)	86 (77)		-
	C, n (%)	21 (23)	20 (23)	1	1.02 (0.53–1.96)

Haplotype analysis showed no relationship between the four haplotypes of ghrelin rs27647 and rs26311 polymorphisms and PE.

The analysis of rs27647 and rs26311 polymorphisms between mild and severe PE showed an association between rs27647 but no rs26311 polymorphism and severe PE (Table 3). The results indicated a relationship between rs27647 and severe PE compared to mild PE and controls. Thus, the rs27647 variant was associated with severe PE compared to mild PE in codominant [OR =1.28 (0.62–2.61) and OR=4.64 (1.47–14.62), p=0.024], recessive [OR =4.22 (1.39–12.84), p=0.008], and log-additive [OR= 1.80 (1.11–2.93), p=0.016] models and controls in codominant [OR= 1.48 (0.75–2.89), and OR=3.39 (1.33–8.65), p=0.036], dominant [OR= 1.85 (1.01–3.39), p=0.048], recessive [OR= 2.94 (1.20–7.21), p=0.020], and log-additive [OR= 1.74 (1.13–2.67), p=0.012] models. No association was observed between rs26311 polymorphism and PE severity. No statistical variation was found between rs27647 and rs26311 polymorphisms and PE in different BMIs (BMI <25 and ≥25).

**Table 3:** Genotypic frequency of maternal ghrelin rs27647 polymorphism in mild and severe preeclampsia.

		Control (n=152)	Mild PE (n=97)	Severe PE (n=59)	p-value	OR (95%CI)
rs27647						
	AA, n (%)	95 (62.5)	59 (60.8)	28 (47.5)		1
	AG, n (%)	46 (30.3)	33 (34)	20 (33.9)		
	GG, n (%)	11 (7.2)	5 (5.2)	11 (18.6)		
Severe PE vs. mild PE						
	Codominant				**0.024**	1.28 (0.62–2.61)
						4.64 (1.47–14.62)
	Dominant (AG + GG vs. AA)				0.10	1.72 (0.89–3.31)
	Recessive (GG vs. AA + AG)				**0.008**	4.22 (1.39–12.84)
	Overdominant (GG+AA vs. AG)				0.99	0.99 (0.50–1.97)
	Log-additive (GG vs. AG vs. AA)				0.016	1.80 (1.11–2.93)
Severe PE vs. control						
	Codominant				**0.036**	1.48 (0.75–2.89)
						3.39 (1.33–8.65)
	Dominant (AG + GG vs. AA)				**0.048**	1.85 (1.01–3.39)
	Recessive (GG vs. AA + AG)				**0.020**	2.94 (1.20–7.21)
	Overdominant (GG+AA vs. AG)				0.610	1.18 (0.62–2.24)
	Log-additive (GG vs. AG vs. AA)				**0.012**	1.74 (1.13–2.67)

Significant p-values are indicated in bold.

The expression of placental ghrelin (mRNA) in the PE group was lower than that in controls (0.67-fold), but the variation did not differ statistically (p=0.263). The placental rs27647 and rs26311 polymorphisms were not related to the ghrelin mRNA expression in the PE and normotensive pregnant women.

## DISCUSSION

In the current investigation, no relation was observed between ghrelin rs27647 and rs26311 variants and PE susceptibility; however, there was a relationship between ghrelin rs27647 polymorphism and severe PE but no mild PE in codominant, recessive, and log-additive models. Although the mRNA expression of placental ghrelin declined in the PE group, this parameter did not differ statistically. In addition, the mRNA expression of placental ghrelin was not different between various genotypes of rs27647 and rs26311 polymorphisms.

In their study, Erol et al. demonstrated that elevated blood ghrelin was related to PE and its severity in pregnancies^19^. Also, Makino et al. examined changes in plasma ghrelin in gestational hypertension, and the results showed that there was a negative relationship between plasma ghrelin and systemic hypertension in healthy pregnant women. In Makino et al.'s study, there were increased ghrelin concentrations in patients with gestational hypertension compared to healthy pregnant women. Moreover, systemic blood pressure was significantly related to plasma ghrelin levels^9^. Also, a study by S. Aydin et al. showed that leptin levels increased in women with PE, especially in those with severe PE. In addition, the leptin levels were elevated in the umbilical venous and arterial sections of the PE group. However, a negative correlation was found between serum ghrelin levels and blood pressure in PE women^12^. Wu et al. found lower ghrelin levels in the serum of PE mothers compared to normal pregnancies^20^. In another study, Zhao-FengLi et al. demonstrated that plasma ghrelin levels were lower in women with gestational hypertension, which was negatively correlated with the mean arterial pressure in them^10^.

In the current study, we found a relationship between ghrelin rs27647 polymorphism and severe PE but not mild PE. To the best of our knowledge, there is no published report on the relationship between ghrelin polymorphisms and PE. However, numerous studies have investigated the effects of ghrelin variants on other diseases such as coronary artery disease (CAD), diabetes, obesity, and metabolic syndrome. In a study conducted by Zhang et al., higher plasma obestatin but not ghrelin levels and small for gestational age (SGA) infants were observed. Moreover, no relationship between ghrelin/obestatin polymorphisms (rs34911341, rs696217, and rs468467) and this complication was observed^21^. In another study, Hedayatizadeh-Omran et al. demonstrated the relationship between ghrelin rs696217 polymorphism (Leu72Met) and heart failure in CAD patients. In addition, they showed higher ghrelin levels in CAD patients and an inverse relationship between ghrelin levels and the rs696217CC genotype. In addition, they found no relationship between ghrelin rs696217 polymorphism and hypertension in CAD patients^22^.

Similar results were obtained from Zhang et al. showing the relation between rs696217 CC genotype and C allele and heart failure in CAD patients^23^. Berthold, H. K. et al. examined the effects of six ghrelin polymorphisms on hypertension and atherosclerotic disease and showed only the effect of rs34911341 Gln51 (Arg51Gln) allele on hypertension susceptibility (2 folds)^24^. Poykko et al. obtained similar results in patients with type 2 diabetes^25^. In a study conducted by ZHU, J. F. et al. on the association between metabolic syndrome and ghrelin Leu72Met polymorphism, no significant relationship was observed^26^. Ozgen et al. showed the effect of A-501C polymorphism on early onset rheumatoid arthritis^27^.

Although this research, for the first time, studied the effects of ghrelin polymorphisms on PE, it had some limitations. For example, the small sample size in the subgroup analysis might affect the present results’ accuracy. Further studies are needed to assay these aspects.

## CONCLUSION

We found no association between maternal and placental ghrelin rs27647 and rs26311 polymorphisms and PE; however, there was an association between ghrelin rs27647 polymorphism and severe PE. The placental ghrelin (mRNA) expression was lower in the PE group, but the variation did not differ statistically. No significant difference was found between the two groups concerning the mRNA expression of the placental ghrelin gene.

## ETHICAL APPROVAL

The study protocol was approved by the Ethics Committee of Zahedan University of Medical Sciences (IR.ZAUMS.REC.1399.428).
